# Occurrence patterns of coral-dwelling gall crabs (Cryptochiridae) over depth intervals in the Caribbean

**DOI:** 10.7717/peerj.1794

**Published:** 2016-03-10

**Authors:** Kaj M. van Tienderen, Sancia E.T. van der Meij

**Affiliations:** 1 Naturalis Biodiversity Center, Leiden, The Netherlands; 2 Oxford University Museum of Natural History, University of Oxford, Oxford, United Kingdom

**Keywords:** Belt transects, Associated fauna, Invertebrates, Coral cover, Scleractinia, Curaçao

## Abstract

Coral-associated invertebrates form a major part of the diversity on reefs, but their distribution and occurrence patterns are virtually unstudied. For associated taxa data are lacking on their distribution across shelves and environmental gradients, but also over various depths. Off Curaçao we studied the prevalence and density of coral-dwelling gall crabs (Cryptochiridae), obligate symbionts of stony corals. Belt transects (10 × 0.5m^2^) were laid out at 6, 12 and 18 m depth intervals at 27 localities. Twenty-one known host coral species were surveyed, measured, and the number of crab dwellings was recorded to study the influence of host occurrence, depth distribution, and colony size on the occurrence rates of three Atlantic gall crab species: *Opecarcinus hypostegus*, *Troglocarcinus corallicola* and *Kroppcarcinus siderastreicola*. The overall gall crab prevalence rate was 20.3% across all available host corals at all depths. The agariciid-associated species *O. hypostegus* was found to mostly inhabit *Agaricia lamarcki* and its prevalence was highest at deeper depths, following the depth distribution of its host. *Kroppcarcinus siderastreicola,* associated with *Siderastrea* and *Stephanocoenia,* inhabited shallower depths despite higher host availability at deeper depths. The generalist species *T. corallicola* showed no clear host or depth specialisation. These results show that the primary factors affecting the distribution and occurrence rates over depth intervals differed between each of the three Atlantic cryptochirid species, which in turn influences their vulnerability to reef degradation.

## Introduction

Coral reefs are amongst the most productive ecosystems on earth and encompass the highest biodiversity of any marine ecosystem. The wealth of potential habitats on reefs has given rise to an enormous diversity of species (Connell, 1978; Sebens, 1994; Gray, 1997; Plaisance et al., 2011). The majority of coral reef biodiversity is composed of highly diverse invertebrate taxa that are understudied and incompletely described (Reaka-Kudla, 1997). A large number of these invertebrates live in close association with scleractinian corals, relying on their hosts for food, refuge and habitat (Stella et al., 2011; Hoeksema, van der Meij & Fransen, 2012). The relationship between coral-associated invertebrates and corals can be either obligate or facultative (Castro, 1976; Castro, 2015). Because obligate species depend on their host coral for survival, their occurrence and distribution is inseparable with that of their host. If particular coral species disappear facultative associates may persist by switching hosts, whereas the more specialized, obligate associates are likely at greater risk of extinction. In turn, many corals are also reliant on the services of particular invertebrate taxa for their subsistence (Stella et al., 2011, and references therein). This high degree of dependency may have severe consequences in light of the continuing global decrease in coral cover, and is likely to cause declines among invertebrates and other coral-associated taxonomic groups. A better understanding of the occurrence patterns of invertebrates and their degree of dependence on their hosts, has thus become an important conservational issue in this era. The presence of coral-associated organisms evidently depends on host availability, which may be related to various environmental factors (e.g., distance offshore, exposure to winds, and depth), but it is yet unclear if and how these factors interact with occurrence rates (Gittenberger & Hoeksema, 2013,van der Meij & Hoeksema, 2013). Unfortunately, very little literature is available on the distribution patterns of coral-associated species, across the shelf or over depths (Preston & Doherty, 1990; Preston & Doherty, 1994; Oigman-Pszczol & Creed, 2006; Gittenberger & Hoeksema, 2013; van der Meij & Hoeksema, 2013).

**Figure 1:**
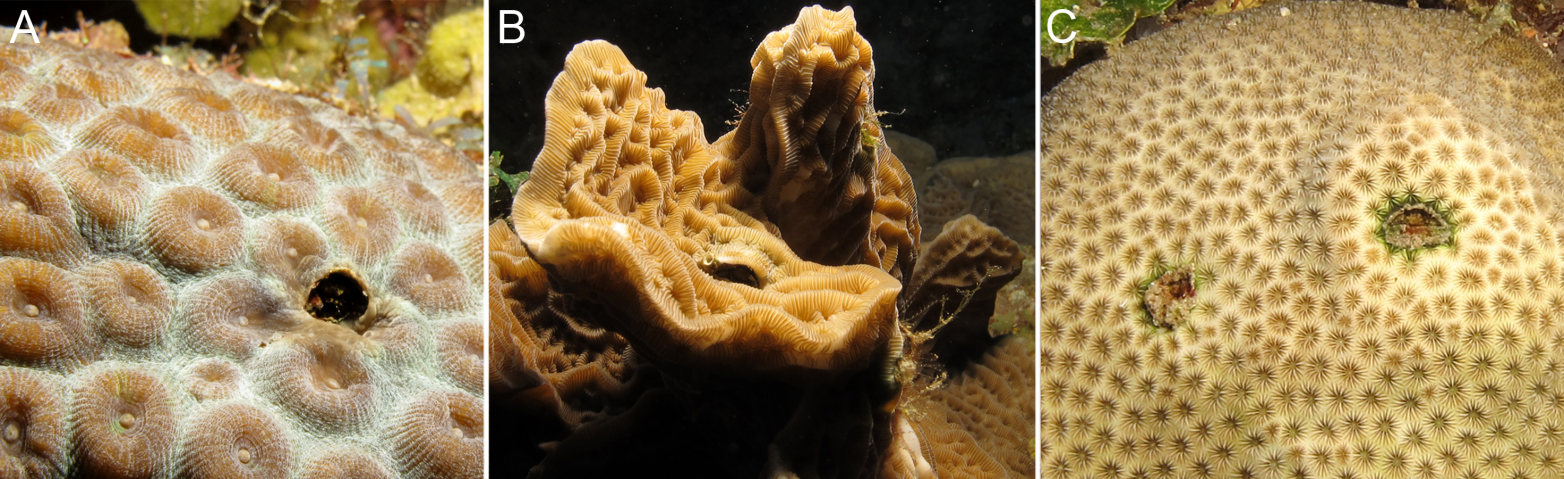
Gall crab dwellings as observed off Curaçao. (A) *Montastraea cavernosa*, (B) *Agaricia agaricites* and (C) *Stephanocoenia intersepta*.

Reef habitats support abundant and diverse assemblages of small crustaceans; a large portion of the more than 500 (out of nearly 2,000) brachyuran crab species on Indo-Pacific coral reefs live in close association with scleractinian corals (Serène, 1972). Among them are coral-dwelling gall crabs (Cryptochiridae), obligate symbionts of stony corals (Scleractinia). These crabs settle on their host coral as megalopae and then modify coral morphology by inducing the growth of pits or galls, which they live in for the rest of their lives (Utinomi, 1944; Castro, 1976; Simon-Blecher & Achituv, 1997; Simon-Blecher et al., 1999; van der Meij, 2012). Gall crabs display a high degree of specificity, mostly at host genus level (Kropp, 1990; van der Meij, 2015), more so in the Indo-Pacific than in the Atlantic (Kropp & Manning, 1987; van der Meij & Nieman, in press). They are common but easily overlooked inhabitants of coral reefs (Hoeksema & van der Meij, 2013). Cryptochirids are an especially interesting group as they can be efficiently studied because of their visibility on the reef surface (Fig. 1), like corals and fish, two taxa that have been the mainstay of reef ecological analyses partly for this reason.

Earlier studies on the occurrence rates of gall crabs were discussed in van der Meij & Hoeksema (2013) and show that gall crab prevalence in host corals ranges between 10 and 37%, but generally around 20% of the host corals are inhabited (Kramarsky-Winter, Galil & Loya, 1995; Simon-Blecher & Achituv, 1997; Carricart-Ganivet et al., 2004; Johnsson et al., 2006; Oigman-Pszczol & Creed, 2006; Nogueira et al., 2014). The observed variation in gall crab occurrence rates within these studies indicates possible links to depth, host species and/or natural and anthropogenic stresses (van der Meij & Hoeksema, 2013). Also, the host colony size may have an effect on the associated fauna composition (Schiemer, Niedermüller & Herler, 2009; Carvalho et al., 2014), and hence host size could also be of influence to the occurrence patterns of cryptochirids. Valid comparisons between studies are, however, hard to be made because of differences in sampling methodology.

The focus of this study was to examine the primary factors affecting the occurrence patterns of three shallow-water Atlantic gall crab species, *Opecarcinus hypostegus* (Shaw & Hopkins, 1977), *Troglocarcinus corallicola* (Verrill, 1908) and *Kroppcarcinus siderastreicola* (Badaro et al., 2012). Each of these gall crab species is associated with distinct host coral families or genera (van der Meij, 2014, and references therein). *Opecarcinus hypostegus* is associated with five Atlantic species of the coral family Agariciidae (Kropp & Manning, 1987; van der Meij, 2014). *Troglocarcinus corallicola* is a generalist that occurs in association with a wide variety of hosts (Kropp & Manning, 1987). Off Curaçao, 14 coral species attributed to four different Atlantic coral families were recorded as hosts for *T. corallicola* by van der Meij (2014). *Kroppcarcinus siderastreicola* is associated with the astrocoeniid *Stephanocoenia intersepta* and members of the Siderastreidae (Badaro et al., 2012; Nogueira et al., 2014; van der Meij, 2014). The level of host specificity in these obligate crab species may have an important effect on their rate of occurrence, depth distribution and vulnerability to local extinction. The mechanisms predicted to influence gall crab occurrence patterns, in addition to the general availability of the host coral species, that were examined in this study, are: (I) associations with particular host species and (II) a restricted depth distribution and (III) host colony surface.

## Materials & Methods

### Data collection

**Figure 2:**
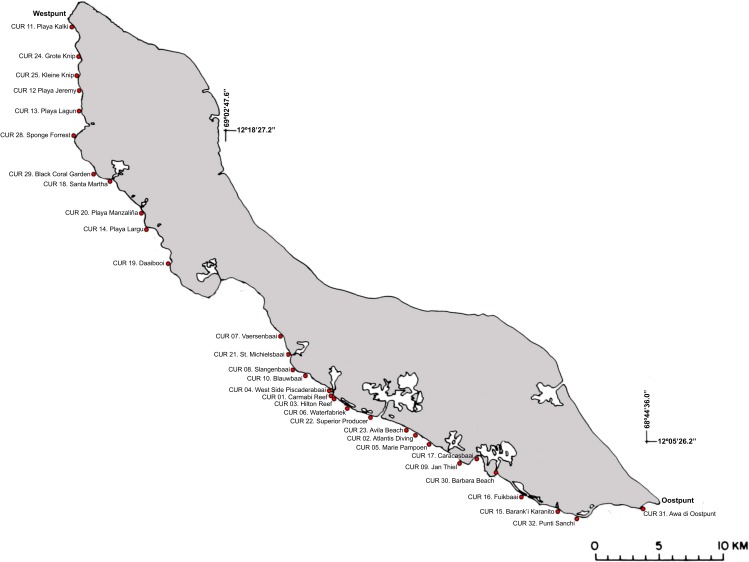
Sampling localities along the leeward side off Curaçao. Detailed locality data is provided in Table S1.

**Figure 3:**
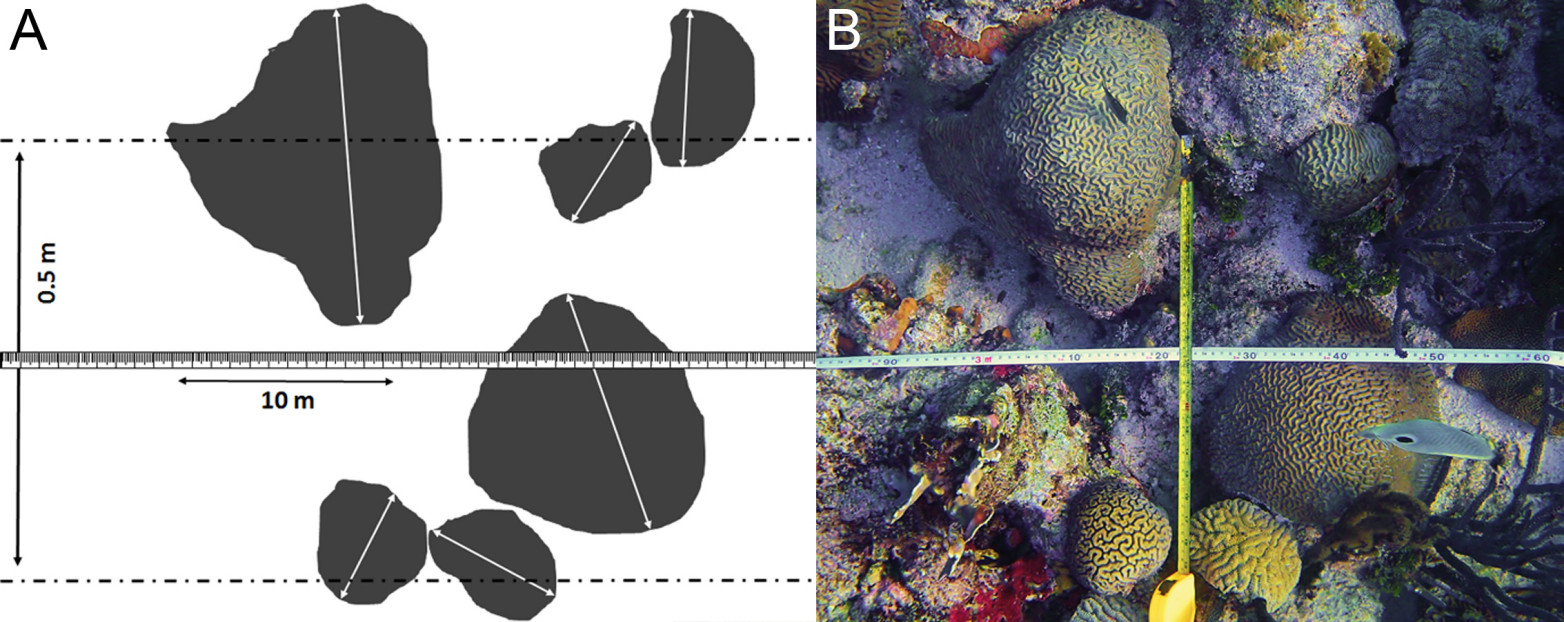
Belt transect survey method used in this study. (A) Schematic view of the belt transect survey method based on (B) the belt transect survey at locality CAR06 at 6 m depth.

Between 12 March and 28 April 2014 a survey was conducted off Curaçao (Dutch Caribbean) in the southern part of the Caribbean Sea. This research was performed under the annual research permit (48584) issued by the Curaçaoan Ministry of Health, Environment and Nature (GMN) to the CARMABI foundation. At 27 localities on the leeward side of the island occurrence patterns and depth distributions of three gall crab species (*Opecarcinus hypostegus*, *Troglocarcinus corallicola* and *Kroppcarcinus siderastreicola*) were examined (Fig. 2 and Table S1). Surveys were conducted using belt transects of 5 m^2^ (10 m × 0.5 m) at three depth intervals (6, 12 and 18 m) at all localities, resulting in a total belt transect survey of 405 m^2^. Within a belt transect all the colonies of coral species listed as host to cryptochirids by van der Meij (2014) were counted, and the diameter (*d*) of the colony was measured at the broadest part (Fig. 3). Many Cryptochiridae host corals in the Caribbean have (sub)massive, hemispherical colonies or consist of flat plates. Coral colonies were therefore considered to be round-shaped, thus colony surface area (*S*) was calculated as *S*=_1∕4_*πd*^2^. For some species this might result in a slight underestimation of colony surface area, e.g., for *Agaricia agaricites* with its upright, bifacial fronds. The number of gall crab dwellings was counted for each individual coral colony. The occurrence rates of the crab dwellings were described using the concepts prevalence and density. Prevalence was determined as the mean proportion of host coral colonies inhabited by gall crabs, whereas density was determined as the mean number of gall crab specimens per 1 m^2^ host colony surface area. Coral density was expressed in surface area (m^2^) or number of colonies (*n*) per transect.

Gall crab specimens were not collected for identification, given the limited time frame during the belt surveys. Based upon the findings by van der Meij (2014)—who sampled approximately 80 lots of gall crabs off Curaçao in 2013 and found absolute host specificity patterns—we attributed all encountered gall crabs in the present study inhabiting Agariciidae to *O. hypostegus*, inhabiting the coral families Meandrinidae, Merulinidae, Montastraeidae or Mussidae to *T. corallicola* and the gall crabs dwelling in Siderastreidae and Astrocoeniidae to *K. siderastreicola*. The corals were visually identified to species level during the surveys using Humann & Deloach (2002), Coralpedia (http://coralpedia.bio.warwick.ac.uk) and the Coral IDC tool (http://www.researchstationcarmabi.org). Coral nomenclature was updated following (Budd et al., 2012), all species authorities are provided in Table 1.

**Table 1:** Overview of the coral species off Curaçao recorded as host to Atlantic cryptochirids based on van der Meij (2014). The total number of encountered colonies, inhabited colonies, dwellings per colony and dwellings per inhabited colony in the present study are provided.

Gall crab species	Coral family	Coral species	Encountered colonies (*n*)	Inhabited colonies (*n*)	Dwellings/ colony	Dwellings/ inhab. colonies
*Opecarcinus hypostegus*	Agariciidae	*Agaricia agaricites* (Linnaeus, 1758)	505	53	0.14	1.34
		*Agaricia fragilis* Dana, 1846	–	–	–	–
		*Agaricia grahamae* Wells, 1973	–	–	–	–
		*Agaricia humilis* Verrill, 1901	44	8	0.18	1.00
		*Agaricia lamarcki* Milne Edwards and Haime, 1851	87	56	0.98	1.55
*Troglocarcinus corallicola*	Meandrinidae	*Dendrogyra cylindrus* Ehrenberg, 1834	−	–	–	–
		*Dichocoenia stokesii* Milne Edwards and Haime, 1848	7	1	0.14	1.00
		*Meandrina meandrites* (Linnaeus, 1758)	90	2	0.02	1.00
	Merulinidae	*Orbicella annularis* (Ellis and Solander, 1786)	332	72	0.32	1.46
		*Orbicella faveolata* (Ellis and Solander, 1786)	106	27	0.37	1.44
		*Orbicella franksi* (Gregory, 1895)	126	48	0.52	1.38
	Montastraeidae	*Montastraea cavernosa* (Linnaeus, 1766)	160	44	0.38	1.39
	Mussidae	*Colpophyllia natans* (Houttuyn, 1772)	60	12	0.27	1.33
		*Diploria labyrinthiformis* (Linnaeus, 1758)	25	3	0.12	1.00
		*Favia fragum* (Esper, 1795)	–	–	–	–
		*Manicina areolata* (Linnaeus, 1758)	–	–	–	–
		*Mussa angulosa* (Pallas, 1766)	–	–	–	–
		*Pseudodiploria clivosa* (Ellis and Solander, 1786)	6	3	0.67	1.33
		*Pseudodiploria strigosa* (Dana, 1846)	65	13	0.48	2.38
*Kroppcarcinus siderastreicola*	Siderastreidae	*Siderastrea siderea* (Ellis and Solander, 1786)	190	33	0.44	2.52
	Astrocoeniidae	*Stephanocoenia intersepta* (Lamarck, 1816)	71	5	0.08	1.20

### Statistical analyses

To determine if there were differences in the coral distribution over the surveyed depths (6, 12 and 18 m) a Friedman test was used. The surface area (m^2^) and no. of colonies (*n*) were compared per coral species over depth intervals at the 27 localities. When a significant difference over depth was obtained, post hoc analyses were run using separate Wilcoxon signed-rank tests, to examine the differences between the depth intervals. To examine whether the observed gall crab prevalence is linked to the general availability of their host corals, a Spearman rank correlation (one-sided) was used to test for a correlation between the number of available host colonies and the number of inhabited host colonies. The influence of depth or host species on the gall crab prevalence was examined using a Chi-square test. When a significant interaction was encountered a post hoc analysis was run to examine the differences in gall crab prevalence between the depth intervals or host species. For each post hoc analysis a Bonferroni correction was applied dividing the significance level of *p* = 0.05 through the number of post hoc tests. All statistical analyses were executed using SPSS Statistics 21 (IBM Corp. in Armonk, NY, 2012). Figures were created with Graphpad Prism v. 5 (GraphPad Software, San Diego, California, USA) to visualize the coral density and gall crab prevalence data, the latter using coenoclines of the mean number of available and inhabited coral colonies within the belt transects.

## Results

Within the total 405 m^2^ of belt transects 1,874 coral colonies were examined, with 380 found to be inhabited. Out of 21 known cryptochirid host species, 15 were encountered. The most abundant coral was *Agaricia agaricites* (*n* = 505), followed by *Orbicella annularis* (*n* = 332) and *Siderastrea siderea* (*n* = 190). In contrast, *Dichocoenia stokesii* (*n* = 7) and *Pseudodiploria clivosa* (*n* = 6) were rarely encountered within the transect surveys and the coral species *Agaricia fragilis*, *A. grahamae*, *Favia fragum*, *Manicina areolata* and *Mussa angulosa* were not observed at all during the present study (Table 1). In total 583 gall crab dwellings (*Opecarcinus hypostegus n* = 166, *Troglocarcinus corallicola n* = 328, *Kroppcarcinus siderastreicola n* = 89) were observed. The highest number of dwellings in a single coral was 24, observed in a colony of *Siderastrea siderea* at 6 m depth. The overall coral cover and diversity in a 10 km^2^ radius from Curaçao’s capital Willemstad was lower than at the other localities, but this difference was not significant.

### Coral depth distribution

The depth distribution was examined for known host corals of cryptochirids off Curaçao in both surface area (m^2^) and number of colonies (*n*). The corals are hereafter grouped according to their associated gall crab species: *Opecarcinus hypostegus* (Agariciidae), *Troglocarcinus corallicola* (Meandrinidae, Merulinidae, Montastraeidae, Mussidae) and *Kroppcarcinus siderastreicola* (*Siderastrea, Stephanocoenia*).

#### Agariciidae

**Figure 4:**
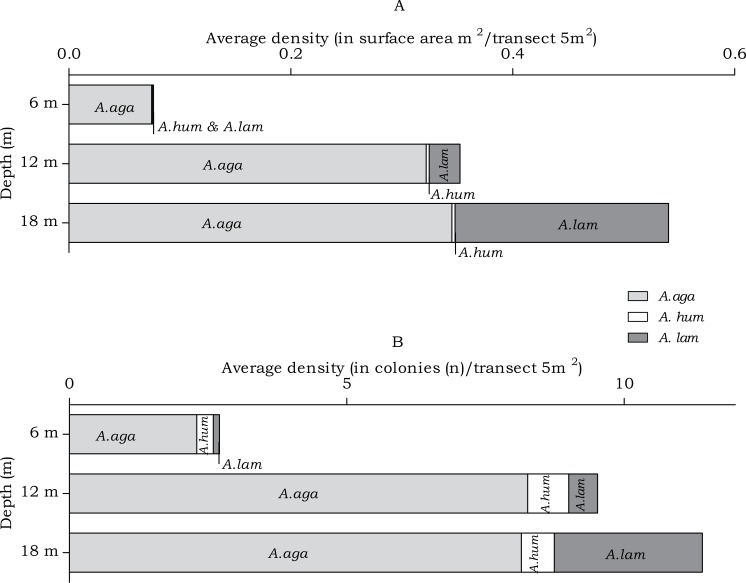
Average Agariciidae coral density off Curaçao. (A) surface area (m^2^) and (B) colonies (*n*) per species per depth interval.

In the belt transects three species of Agariciidae were encountered: *Agaricia agaricites, A. humilis* and *A. lamarcki.* The abundance of the agariciid species differed over the three depth intervals in both surface area (m^2^) and no. of colonies (*n*) (Fig. 4). Post hoc tests revealed a significant increase from 6 m to 12 m depth and from 6 m to 18 m depth (Table 2). Distinct depth distributions were found for the three agariciid species encountered in this study. At all depth intervals *Agaricia agaricites* was the most dominant species (Fig. 4). An increase in the *A. agaricites* surface area and number of colonies was found over increasing depth. Post hoc tests revealed this increase was significant for the surface area and number of colonies from 6 m to 12 m depth. From 6 m to 18 m depth the increase was only significant for the surface area (Table 2). *Agaricia lamarcki* occurred at 6 m in very low abundance, becoming significantly more abundant with increasing depth (Table 2 and Fig. 4). Post hoc tests revealed a significant increase from 6 to 12 m, from 6 to 18 m and from 12 to 18 m depth, except for the number of colonies between 6 and 12 m (Table 2). *Agaricia humilis* occurred at all depths in low abundances and colony sizes were small compared to *A. agaricites* and *A. lamarcki* (Fig. 4).

**Table 2:** Statistical differences in Agariciidae density between depth intervals.

Coral species	Friedman test	Post Hoc tests		
		6 m–12 m	6 m–18 m	12 m–18 m
All species surface area (m^2^)	*X*^2^(2) = 22.824, *p* < 0.001^*^	*Z* = − 3.371, *p* < 0.001^**^	*Z* = − 3.721, *p* < 0.001^**^	*Z* = − 1.384, *p* = 0.173
All species colonies (*n*)	*X*^2^(2) = 19.146, *p* < 0.001^*^	*Z* = − 3.424, *p* < 0, 001^**^	*Z* = − 3.579, *p* < 0.001^**^	*Z* = − 0.315, *p* = 0.763
*A. agaricites* surface area (m^2^)	*X*^2^(2) = 11.043, *p* = 0.003^*^	*Z* = − 3.036, *p* = 0.001^**^	*Z* = − 2.971, *p* = 0.002^**^	*Z* = − 0.276, *p* = 0.799
*A. agaricites* colonies (*n*)	*X*^2^(2) = 11.890, *p* = 0.002^*^	*Z* = − 3.200, *p* = 0.001^**^	*Z* = − 2.304, *p* = 0.020	*Z* = − 0.439, *p* = 0.673
*A. lamarcki* surface area (m^2^)	*X*^2^(2) = 25.914, *p* < 0.001^*^	*Z* = − 2.701, *p* = 0.004^**^	*Z* = − 3.724, *p* < 0.001^**^	*Z* = − 3.042, *p* = 0.001^**^
*A. lamarcki* colonies (*n*)	*X*^2^(2) = 24.471, *p* < 0.001^*^	*Z* = − 2.326, *p* = 0.027	*Z* = − 3.631, *p* < 0.001^**^	*Z* = − 3.287, *p* < 0.001^**^
*A. humilis* surface area (m^2^)	*X*^2^(2) = 7.536, *p* = 0.0210^*^	*Z* = − 2.342, *p* = 0.016	*Z* = − 1.600, *p* = 0.123	*Z* = − 0.543, *p* = 0.605
*A. humilis* colonies (*n*)	*X*^2^(2) = 4.509, *p* = 0.1100	–	–	–

**Notes.**

* Indicates statistical significance (*p* = 0.05).

** Indicates statistical significance after Bonferroni correction, applied for the post hoc tests (*p* = 0.0125).

#### Meandrinidae, Merulinidae, Montastraeidae, Mussidae

**Figure 5:**
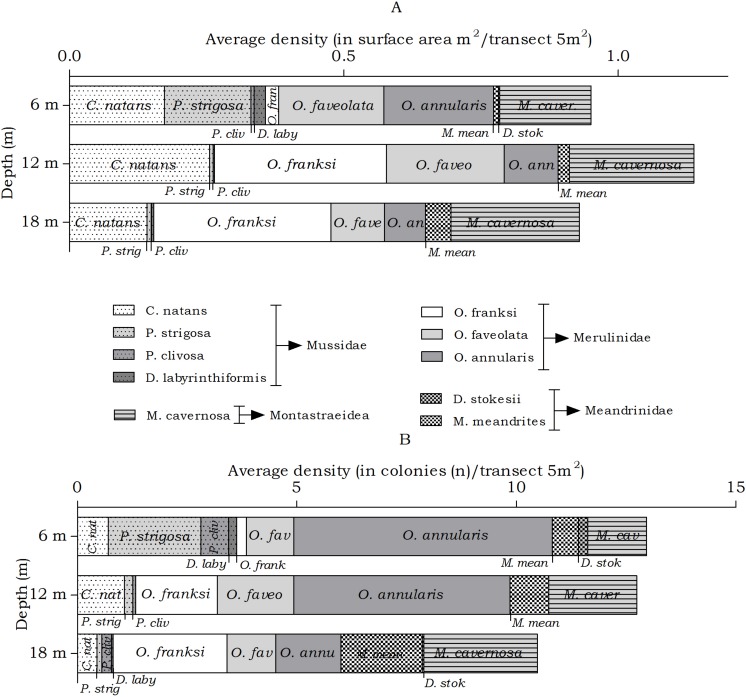
Average coral density (Meandrinidae, Merulinidae, Montastraeidae, Mussidae) off Curaçao. (A) surface area (m^2^) and (B) colonies (*n*) per species per depth interval.

The abundance of the host corals of *Troglocarcinus corallicola* did not statistically differ over depth in surface area (m^2^) or number of colonies (*n*), but did reveal differences in the distribution ranges of these coral species. At all depths members of the Merulinidae family were the most dominant. *Orbicella annularis* and *O. faveolata* showed similar density (in m^2^) at the 6 m interval, but not in number of colonies. *Orbicella annularis* colonies were more numerous but smaller than those of *O. faveolata.* For the genus, *O. franksi* had the highest surface area at the 12 and 18 m intervals (Fig. 5). Significant differences over depth were only found for *O. franksi, Pseudodiploria strigosa, Meandrina meandrites* and *Diploria labyrinthiformis* (Table 3), yet at all depth intervals *D. labyrinthiformis* was relatively rare. *Pseudodiploria strigosa* has a distinct shallow (6 m) distribution, whereas *M. meandrites* becomes more abundant with depth. *Colpophyllia natans* and *Montastraea cavernosa* are rather dominant at all depths (Fig. 5).

**Table 3:** Statistical differences in coral density of Meandrinidae, Merulinidae, Montastraeidae, and Mussidae between depth intervals.

Coral species	Friedman test	Post Hoc tests		
		6 m–12 m	6 m–18 m	12 m–18 m
All species surface area (m^2^)	*X*^2^(2) = 0.519, *p* = 0.826	–	–	–
All species colonies (*n*)	*X*^2^(2) = 0.585, *p* = 0.753	–	–	–
*Colpophyllia natans* surface area (m^2^)	*X*^2^(2) = 0.794, *p* = 0.695	–	–	–
*Colpophyllia natans* colonies (*n*)	*X*^2^(2) = 1.733, *p* = 0.430	–	–	–
*Pseudodiploria strigosa* surface area (m^2^)	*X*^2^(2) = 17.370, *p* < 0.001^*^	*Z* = − 3.124, *p* = 0.001^**^	*Z* = − 2.638, *p* = 0.006^**^	*Z* = − 0.105, *p* = 1.000
*Pseudodiploria strigosa* colonies (*n*)	*X*^2^(2) = 16.302, *p* < 0.001^*^	*Z* = − 2.991, *p* = 0.001^**^	*Z* = − 3.134, *p* = 0.001^**^	*Z* = − 0.649, *p* = 0.531
*Pseudodiploria clivosa* surface area (m^2^)	*X*^2^(2) = 5.200, *p* = 0.136	–	–	–
*Pseudodiploria clivosa* colonies (*n*)	*X*^2^(2) = 5.200, *p* = 0.136	–	–	–
*Diploria labyrinthiformis* surface area (m^2^)	*X*^2^(2) = 6.533, *p* = 0.039	*Z* = − 1.956, *p* = 0.054	*Z* = − 1.804, *p* = 0.077	*Z* = − 0.772, *p* = 0.477
*Diploria labyrinthiformis* colonies (*n*)	*X*^2^(2) = 6.045, *p* = 0.047	*Z* = − 2.438, *p* = 0.016	*Z* = − 2.047, *p* = 0.045	*Z* = − 1.414, *p* = 0.289
*Orbicella franksi* surface area (m^2^)	*X*^2^(2) = 12.824, *p* = 0.001^*^	*Z* = − 2.769, *p* = 0.004^**^	*Z* = − 2.760, *p* = 0.003^**^	*Z* = − 0.201, *p* = 0.860
*Orbicella franksi* colonies (*n*)	*X*^2^(2) = 15.631, *p* < 0.001^*^	*Z* = − 3.069, *p* = 0.001^**^	*Z* = − 3.062, *p* < 0.001^**^	*Z* = − 0.920, *p* = 0.371
*Orbicella faveolata* surface area (m^2^)	*X*^2^(2) = 0.102, *p* = 0.969	–	–	–
*Orbicella faveolata* colonies (*n*)	*X*^2^(2) = 0.102, *p* = 0.969	–	–	–
*Orbicella annularis* surface area (m^2^)	*X*^2^(2) = 4.667, *p* = 0.102	–	–	–
*Orbicella annularis* colonies (*n*)	*X*^2^(2) = 5.935, *p* = 0.052	–	–	–
*Dichocoenia stokesii* surface area (m^2^)	*X*^2^(2) = 5.600, *p* = 0.111	–	–	–
*Dichocoenia stokesii* colonies (*n*)	*X*^2^(2) = 4.667, *p* = 0.222	–	–	–
*Meandrina meandrites* surface area (m^2^)	*X*^2^(2) = 9.851, *p* = 0.007^*^	*Z* = − 2.053, *p* = 0.039	*Z* = − 3.431, *p* < 0.001^**^	*Z* = − 2.286, *p* = 0.021
*Meandrina meandrites* colonies (*n*)	*X*^2^(2) = 11.725, *p* = 0.002^*^	*Z* = − 1.543, *p* = 0.165	*Z* = − 3.003, *p* = 0.001^**^	*Z* = − 2.429, *p* = 0.013
*Montastraea cavernosa* surface area (m^2^)	*X*^2^(2) = 5.267, *p* = 0.073	–	–	–
*Montrastraea cavernosa* colonies (*n*)	*X*^2^(2) = 5.886, *p* = 0.053	–	–	–

**Notes.**

* Indicates statistical significance (*p* = 0.05).

** Indicates statistical significance after Bonferroni correction, applied for the post hoc tests (*p* = 0.0125).

**Figure 6:**
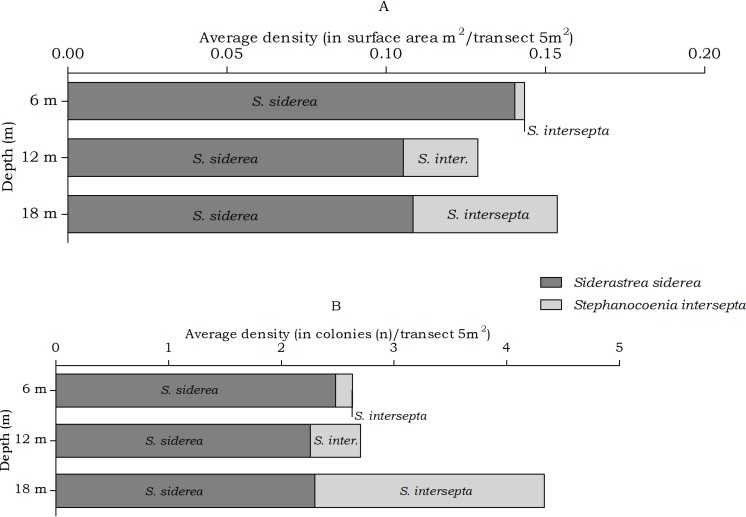
Average coral density of *Siderastrea* and *Stephanocoenia* off Curaçao. (A) surface area (m^2^) and (B) colonies (*n*) per species per depth interval.

**Table 4:** Statistical differences in *Siderastrea siderea* and *Stephanocoenia intersepta* density between depth intervals.

Coral species	Friedman test	Post Hoc tests		
		6 m–12 m	6 m–18 m	12 m–18 m
All species surface area (m^2^)	*X*^2^(2) = 5.057, *p* = 0.082	–	–	–
All species colonies (*n*)	*X*^2^(2) = 12.733, *p* = 0.001^*^	*Z* = − 0.123, *p* = 0.911	*Z* = − 1.999, *p* = 0.045	*Z* = − 2.113, *p* = 0.034
*S. siderea* surface area (m^2^)	*X*^2^(2) = 1.021, *p* = 0.620	–	–	–
*S. siderea* colonies (*n*)	*X*^2^(2) = 1.717, *p* = 0.433	–	–	–
*S. intersepta* surface area (m^2^)	*X*^2^(2) = 23.172, *p* < 0.001^*^	*Z* = − 1.379, *p* = 0.182	*Z* = − 3.823, *p* < 0.001^**^	*Z* = − 3.150, *p* = 0.001^**^
*S. intersepta* colonies (*n*)	*X*^2^(2) = 26.964, *p* < 0.001^*^	*Z* = − 1.732, *p* = 0.148	*Z* = − 4.116, *p* < 0.001^**^	*Z* = − 3.553, *p* < 0.001^**^

**Notes.**

* Indicates statistical significance (*p* = 0.05).

** Indicates statistical significance after Bonferroni correction, applied for the post hoc tests (*p* = 0.0125).

#### Siderastrea, Stephanocoenia

The abundance of the coral species *Siderastrea siderea* and *Stephanocoenia intersepta*, host to *Kroppcarcinus siderastreicola,* differed statistically over depth in number of colonies, but post hoc tests showed no significant difference between the depth intervals (Table 4). At the three depths *S. siderea* is the more dominant species (Fig. 6). *Stephanocoenia intersepta* abundances significantly differed in surface area and number of colonies. Post hoc tests revealed a significant increase in abundance from 6 m to 18 m depth as well as from 12 m to 18 m depth (Table 4). At 18 m depth the number of *S. intersepta* colonies is roughly equal to that of *S. siderea* (Fig. 6B), with *S. siderea colonies* being larger in size.

### Gall crab occurrence rates

#### Opecarcinus hypostegus

**Figure 7:**
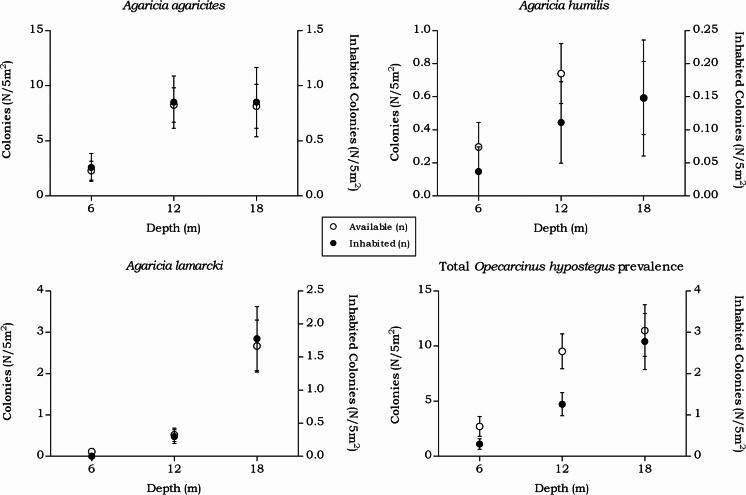
Coenoclines of the mean number of available and inhabited agariciid colonies at the 6, 12 and 18 m depth intervals. Whiskers show standard error. Significant positive correlations between the number of available colonies (*n*) and number of inhabited colonies (*n*) were found for all agariciids. With the strongest correlation found for *A. lamarcki* (*R*_*s*_ = 0.892, *p* < 0.001), followed by a moderate correlation for *A. agaricites* (*R*_*s*_ = 0.662, *p* < 0.001) and *A. humilis* (*R*_*s*_ = 0.491, *p* < 0.001). The overall correlation coefficient between the total available and inhabited hosts was strong (*R*_*s*_ = 0.757, *p* < 0.001).

The prevalence and density of *O. hypostegus* differed over depth for each agariciid coral (Table 5 and Fig. 7). The prevalence of this species was highest at 18 m depth where just below 25% of the available hosts were inhabited, whilst at 6 m depth 14% of the agariciid corals was inhabited. In contrast, the mean *O. hypostegus* density (n/m^2^) was greatest at 6 m and was lowest at 18 m depth. *Opecarcinus hypostegus* shows high density numbers in *Agaricia humilis* at all depth intervals, but these are linked to the small size of the colonies (Fig. 4). The prevalence at 6 m depth were the highest in *A. agaricites* (19.7 %), but at 12 and 18 m the highest prevalence was found in *A. lamarcki* (Table 5). A significant interaction was measured between prevalence and the depth intervals (*X*(2) = 14.568, *p* = 0.001). Post hoc tests revealed a difference in the *O. hypostegus* prevalence between 6 m and 18 m depth (*X*(1) = 6.211, *p* = 0.017) as well as between 12 m and 18 m depth (*X*(1) = 11.128, *p* = 0.001). A significant interaction was also measured between the *O. hypostegus* prevalence and the host species (*X*(2) = 138.872, *p* < 0.001). Post hoc tests revealed a difference in prevalence between *A. agaricites* and *A. lamarcki* (*X*(1) = 138.803, *p* < 0.001) and between *A. humilis* and *A. lamarcki* (*X*(1) = 23.607, *p* < 0.001).

**Table 5:** *Opecarcinus hypostegus* occurrence variables over depth intervals. The total number of host colonies (Col. total) and the mean number of host colonies per transect (Col. mean), with the total and mean number of encountered dwellings between the brackets. Also, the total *O. hypostegus* prevalence (% Inh.) and mean density (n/m^2^) per transect are provided.

	6 m				12 m				18 m			
Coral species	Col. total	Col. mean	% Inh.	Density	Col. total	Col. mean	% Inh.	Density	Col. total	Col. mean	% Inh.	Density
All species	73 (9)	2.70 (0.33)	14.0	28.24	257 (47)	9.52 (1.74)	16.6	8.81	308 (110)	11.41 (4.07)	23.7	7.85
*Agaricia agaricites*	62 (8)	2.30 (0.30)	19.7	3.92	223 (32)	8.26 (1.19)	9.2	5.96	220 (31)	8.15 (1.15)	8.4	1.84
*Agaricia humilis*	8 (1)	0.30 (0.04)	8.3	81.06	20 (3)	0.74 (0.11)	18.0	42.26	16 (4)	0.59 (0.15)	23.8	88.42
*Agaricia lamarcki*	3 (0)	0.11 (0.00)	0.0	0.00	14 (12)	0.52 (0.44)	59.3	25.03	72 (75)	2.67 (2.78)	66.9	18.08

#### Troglocarcinus corallicola

**Figure 8:**
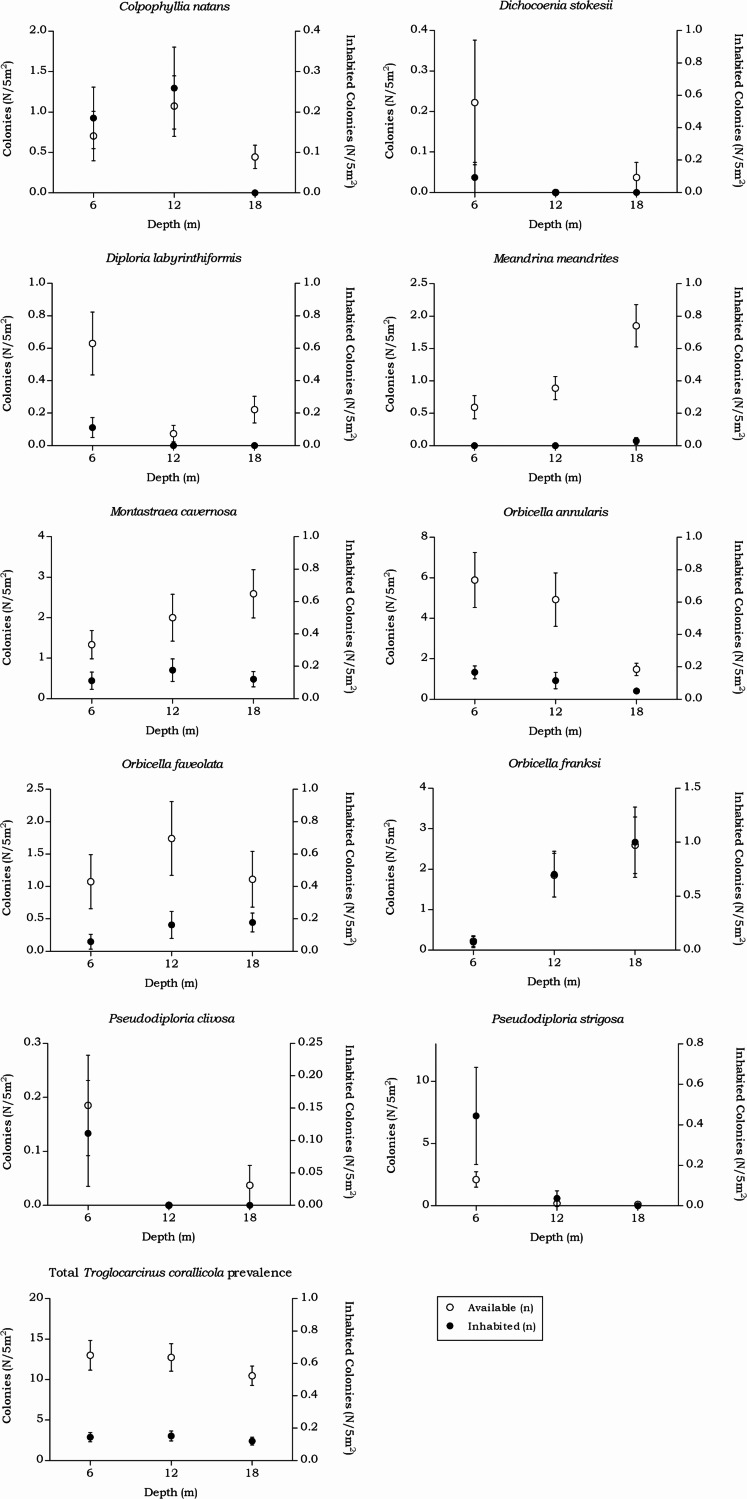
Coenoclines of the mean number of available and inhabited Meandrinidae, Merulinidae, Montastraeidae and Mussidae colonies at the 6, 12 and 18 m depth intervals. Whiskers show standard error. Significant positive correlations between the number of available colonies (*n*) and number of inhabited colonies (*n*) were found for all host species, except for *M. meandrites* (*R*_*s*_ = 0.151, *p* = 0.089). The strongest correlations were found for members of the Merulinidae family (*O. franksi R*_*s*_ = 0.792, *p* < 0.001, *O. faveolata R*_*s*_ = 0.723, *p* < 0.001 and *O. annularis R*_*s*_ = 0.722, *p* < 0.001), followed by moderate correlations for *M. cavernosa* (*R*_*s*_ = 0.652, *p* < 0.001), *P. clivosa* (*R*_*s*_ = 0.633, *p* < 0.001), *P. strigosa* (*R*_*s*_ = 0.572, *p* < 0.001), *D. stokesii* (*R*_*s*_ = 0.484, *p* < 0.001), *C. natans* (*R*_*s*_ = 0.413, *p* < 0.001) and *D. labyrinthiformis* (*R*_*s*_ = 0.413, *p* < 0.001). The overall correlation between the total available and inhabited host colonies was strong (*R*_*s*_ = 0.720, *p* < 0.001).

The prevalence in all hosts across the depth intervals was stable around 20%. The density showed a small decrease over increasing depth from 7.51 to 5.12 (n/m^2^). The *T. corallicola* prevalence and density rates found in the corals species separately did differ over depth (Table 6 and Fig. 8). No significant interaction was measured between the *T. corallicola* prevalence and the various depth intervals (*X*(2) = 0.236, *p* = 0.891). However, the interaction between the *T. corallicola* prevalence and the host species was significant (*X*(9) = 45.912, *p* < 0.001). Post hoc tests revealed a significant difference between the prevalence rate in *M. meandrites* and in all other coral species (*X*(1), *p* ≤ 0.001), except for the lesser abundant coral species *D. stokesii* and *D. labyrinthiformis*. Furthermore, a significant difference in prevalence was found between *O. franksi* and *O. annularis* (*X*(1) = 12.718, *p* = 0.001).

**Table 6:** *Troglocarcinus corallicola* occurrence variables over depth intervals. The total number of host colonies (Col. total) and the mean number of host colonies per transect (Col. mean), with the total and mean number of encountered dwellings between the brackets. Also, the total *O. hypostegus* prevalence (% Inh.) and mean density (n/m^2^) per transect are provided.

	6 m				12 m				18 m			
Coral species	Col. total	Col. mean	% Inh.	Density	Col. total	Col. mean	% Inh.	Density	Col. total	Col. mean	% Inh.	Density
All species	351 (130)	13.00 (4.81)	20.4	7.51	344 (111)	12.74 (4.11)	22.2	6.52	283 (87)	10.48 (3.22)	21.4	5.12
*Colpophyllia natans*	19 (8)	0.70 (0.30)	40.1	21.22	29 (8)	1.07 (0.30)	27.5	4.32	12 (0)	0.44 (0.00)	0.0	0.00
*Dichocoenia stokesii*	6 (1)	0.22 (0.04)	33.3	6.48	0 (0)	0.00 (0.00)	–	–	1 (0)	0.04 (0.00)	0.0	0.00
*Diploria labyrinthiformis*	17 (3)	0.63 (0.11)	20.4	12.46	2 (0)	0.07 (0.00)	0.0	0.00	6 (0)	0.22 (0.00)	0.0	0.00
*Meandrina meandrites*	16 (0)	0.59 (0.00)	0.0	0.00	24 (0)	0.89 (0.00)	0.0	0.00	50 (2)	1.85 (0.07)	6.0	2.37
*Montastraea cavernosa*	36 (19)	1.33 (0.70)	26.5	7.00	54 (27)	2.00 (1.00)	38.8	6.45	70 (15)	2.59 (0.55)	12.6	1.51
*Orbicella annularis*	159 (55)	5.89 (2.04)	22.7	70.8	133 (31)	4.93 (1.15)	12.7	7.01	40 (19)	1.48 (0.70)	32.2	13.95
*Orbicella faveolata*	29 (7)	1.07 (0.26)	7.3	1.11	47 (17)	1.74 (0.63)	16.4	4.35	30 (15)	1.11 (0.55)	64.4	12.52
*Orbicella franksi*	6 (3)	0.22 (0.11)	27.8	4.82	50 (27)	1.85 (1.00)	51.6	17.83	70 (36)	2.59 (1.33)	43.0	9.32
*Pseudodiploria clivosa*	5 (4)	0.19 (0.15)	50.0	14.74	0 (0)	0.00 (0.00)	–	–	1 (0)	0.04 (0.00)	0.0	0.00
*Pseudodiploria strigosa*	57 (30)	2.11 (1.11)	13.1	7.39	5 (1)	0.19 (0.04)	16.7	13.61	3 (0)	0.11 (0.00)	0.0	0.00

#### Kroppcarcinus siderastreicola

**Figure 9:**
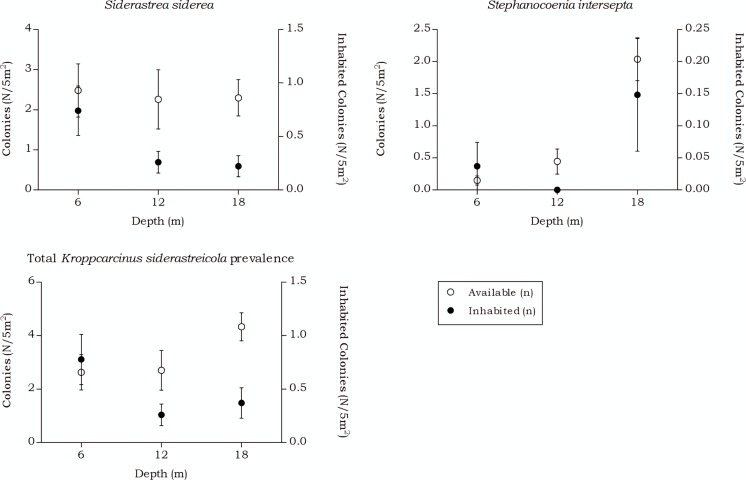
Coenoclines of the mean number of available and inhabited *Siderastrea siderea* and *Stephanocoenia intersepta* colonies at the 6, 12 and 18 m depth intervals. Whiskers show standard error. Significant positive correlations were found between the number of available colonies (*n*) and number of inhabited colonies (*n*) for both coral species, with a moderate correlation coefficient for *S. siderea* (*R*_*s*_ = 0.558, *p* < 0.001) and a weak correlation coefficient for *Stephanocoenia intersepta* (*R*_*s*_ = 0.312, *p* = 0.002). The overall correlation coefficient between the total available and inhabited hosts was moderate (*R*_*s*_ = 0.470, *p* < 0.001).

Over all depth intervals, the highest *K. siderastreicola* prevalence was found in the host coral *Siderastrea siderea* and its prevalence and density was highest at 6 m depth (Table 7 and Fig. 9). A significant interaction was measured between the *K. siderastreicola* prevalence and the depth intervals (*X*(2) = 17.723, *p* < 0.001). Post hoc tests revealed a difference in the *K. siderastreicola* prevalence between 6 m and 12 m depth (*X*(1) = 9.181, *p* = 0.003) as well as between 6 m and 18 m depth (*X*(1) = 14.192, *p* < 0.001). However, no difference in *K. siderastreicola* prevalence was found between 12 m and 18 m depth (*X*(1) = 0.060, *p* = 1.000). A significant difference in *K. siderastreicola* prevalence was also measured between the coral species (*X*(1) = 4.430, *p* = 0.047).

**Table 7:** *Kroppcarcinus siderastreicola* occurrence variables over depth intervals. The total number of host colonies (Col. total) and the mean number of host colonies per transect (Col. mean), with the total and mean number of encountered dwellings between the brackets. Also, the total *K. siderastreicola* prevalence (% Inh.) and mean density (n/m^2^) per transect are provided.

	6 m				12 m				18 m			
Coral species	Col. total	Col. mean	% Inh.	Density	Col. total	Col. mean	% Inh.	Density	Col. total	Col. mean	% Inh.	Density
All species	71 (60)	2.63 (2.22)	29.8	35.01	73 (14)	2.70 (0.52)	10.9	17.07	117 (15)	4.33 (0.56)	8.3	3.77
*Siderastrea siderea*	67 (59)	2.48 (2.19)	33.1	38.15	61 (14)	2.26 (0.52)	16.1	21.94	62 (10)	2.30 (0.37)	10.1	5.31
*Stephanocoenia intersepta*	4 (1)	0.15 (0.04)	25.0	40.53	12 (0)	0.44 (0.00)	0.0	0.00	55 (5)	2.04 (0.19)	6.7	4.07

## Discussion

### Coral depth distribution

In the belt transects we found that for the Agariciidae, *Agaricia agaricites* was the dominant species in surface area and number of colonies at all three transect depths followed by *A. lamarcki* and *A. humilis*. The total Agariciidae abundance, as well as the abundance of all three agariciid species encountered within the belt transects, showed an increase over increasing depth and distinct depth distributions were retrieved for the three *Agaricia* species (Fig. 4), which is in agreement with Bongaerts et al. (2013). For some of the host corals of *Troglocarcinus corallicola* distinct depth distributions were observed (Fig. 5). *Pseudodiploria strigosa* was a rather abundant species at 6 m, but was rarely encountered at 12 or 18 m depth, whereas *Orbicella franksi* was rarely observed at 6 m, but occurred in high density at 12 and 18 m depth. Weil & Knowlton (1994) showed earlier that *Orbicella* spp., like *Agaricia* spp., have distinct depth zonations. Although less pronounced, an increase in density over depth was also observed for *Meandrina meandrites* and a decrease in density over depth was revealed for the relatively rare (in the belt transects) *Diploria labyrinthiformis*. In turn, and similar to the total *T. corallicola* host coral density, *Colpophyllia natans, Orbicella annularis, O. faveolata, Pseudodiploria clivosa, Dichocoenia stokesii* and *Montastraea cavernosa* abundances did not show differences over depth intervals (Fig. 5). For *Siderastrea siderea* no distinct depth zonation was observed, whereas *Stephanocoenia intersepta* became more abundant with increasing depth.

### Gall crab occurrence rates

The distinct depth distributions retrieved in this study directly impact the host-specific obligate symbiont fauna. The occurrence rates of *Opecarcinus hypostegus*, strictly associated with Agariciidae corals, followed the availability of the host and highest prevalence changed from *A. agaricites* to *A. lamarcki* with increasing depth (Table 5 and Fig. 7). *Agaricia lamarcki* is known to be the dominant agariciid species beyond 25 m depth off Curaçao (Bongaerts et al., 2010; Bongaerts et al., 2013). The associated gall crab’s depth distribution is known to extend to at least 60 m depth (van der Meij, van Tienderen & Hoeksema, 2015), and possibly further based on the host’s depth distribution (Bongaerts et al., 2013).

The total available host coral density for *Troglocarcinus corallicola,* a generalist species associated with the coral families Mussidae, Merulinidae, Meandrinidae and Montastraeidae, did not significantly differ between the recorded depths in this study (Fig. 5). The occurrence rates of *T. corallicola* were found affected by neither depth nor host species, and prevalence was stable at about 20%. Although not statistically significant, higher prevalence rates were observed in coral species belonging to the most dominant host coral family Merulinidae at 12 and 18 m depths and within this family, *Orbicella franksi* was significantly more inhabited than *O. annularis*. In addition, the coral species *Meandrina meandrites* was significantly less inhabited than any of the other host corals, except for the least encountered coral species in the present study *Dichocoenia stokesii* and *Diploria labyrinthiformis* (Table 6). This could indicate a (yet unknown) disadvantage for inhabiting *M. meandrites* compared to settling in one of the other host coral species. The answer may also lie in the large phylogenetic distance that was reported between Meandrinidae species and corals from the other host coral families Merulinidae, Mussidae and Montastraeidae (Fukami et al., 2008). The tight evolutionary relationship between corals and gall crabs (Kropp, 1990; van der Meij & Reijnen, 2014; van der Meij, 2015) suggests that *T. corallicola*, which more commonly inhabits Merulinidae, Mussidae and Montastraeidae corals, might be less adapted to inhabiting coral species belonging to the phylogenetically distant Meandrinidae family (including *M. meandrites* and *D. stokesii*).

The occurrence rates of the gall crab species *Kroppcarcinus siderastreicola*, associated with *Siderastrea siderea* and *Stephanocoenia intersepta* off Curacao, were found to be mainly affected by depth. The total available host coral density for *K. siderastreicola* did not significantly differ between the recorded depths in this study nor did the density of its most common host species *S. siderea* (Fig. 6). *Stephanocoenia intersepta*, however, did show variation in density over depth with a significant increase in abundance over increasing depth (Fig. 6 and Table 4). Despite the statistically equal host availability over depth, the *K. siderastreicola* occurrence rate decreased with increasing depth. Settling *K. siderastreicola* larvae exhibit a tendency towards shallower water (Table 7).

In the Atlantic, a prevalence of ca. 20% has now been recovered for gall crabs on three separate occasions. Apart from this study, in the Mexican Caribbean 21% of *Manicina areolata* corals (*n* = 160) were inhabited by *Troglocarcinus corallicola* (Carricart-Ganivet et al., 2004) and 21% of the *Siderastrea stellata* colonies were inhabited by *K. siderastreicola* in Brazil (Nogueira et al., 2014).

### Diversity, distribution and vulnerability

The majority of the observed dwellings were attributed to generalist species *Troglocarcinus corallicola*, followed by *Opecarcinus hypostegus* and *Kroppcarcinus siderastreicola.* For all corals on average 1.42 dwellings per inhabited coral colony were encountered (Table 1). *Kroppcarcinus siderastreicola* averaged 1.86 dwellings per colony, which is comparable to the average of two dwellings per host coral (*Siderastrea stellata*) found by Nogueira et al. (2014).

Following from the intimate relationship between associated taxa and corals, what benefits the coral will ultimately benefit the associate fauna (Oigman-Pszczol & Creed, 2006). The presence and abundance of infaunal species increases with increasing coral cover and number of (branching) coral colonies (Cantera et al., 2003). Local environmental conditions which favour coral distribution and composition could therefore also have a positive effect on the settlement of associates. Between Piscadera Baai (Piscadera Bay) and Willemstad (Fig. 2) coral diversity and cover was lower than in the rest of Curaçao, and the gall crab prevalence dropped from about 20% to 13%. The bay is known to be contaminated with sewage effluent overflow (Nagelkerken, 2006). Off Curaçao, Cryptochiridae are more common in non-disturbed areas, which is in line with results for the Indo-Pacific (van der Meij & Hoeksema, 2013). Such findings make cryptochirids, and possibly other coral-associated taxa, suitable as environmental indicators. A more complete description of the diversity and distribution of coral-associated invertebrates is needed to understand the roles they play in coral reef ecosystems (Stella et al., 2011).

The observed differences in the primary factors affecting the occurrence patterns of the three gall crab species in this study could influence their vulnerability to environmental change. Curaçaoan reefs—and reefs in the wider Caribbean—have been in decline for at least 40 years (Gardner et al., 2003; Bak, Nieuwland & Meesters, 2005; Nagelkerken et al., 2005; Vermeij et al., 2011). Between 1973 and 2002 no significant decline in the cover of *Agaricia* species was found, but the cover of other species, mostly those with non-flat, hemispherical colony surfaces such as *Pseudodiploria strigosa, Stephanocoenia intersepta,* and *Colpophyllia natans*, decreased with time (Bak, Nieuwland & Meesters, 2005). Contrary to this study, a decline in cover of *Agaricia* spp. off Curaçao was reported by (Nagelkerken et al., 2005). Juvenile coral abundance has strongly decreased for Agariciidae (Vermeij et al., 2011). Specialists species *Opecarcinus hypostegus* and *Kroppcarcinus siderastreicola* have a narrow range of inhabitable host corals, and hence may be more vulnerable to current and future rates of coral cover decline than the generalist cryptochirid species *Troglocarcinus corallicola. Opecarcinus hypostegus*’ tendency to inhabit deeper waters, however, has an advantage over the shallow water tendency found for *K. siderastreicola*, because in contrast to the declining coral cover in the shallow reefs (10–20 m) no noticeable decrease was detected in the deep reef cover (30–40 m) over 30 years’ time off Curaçao (Bak, Nieuwland & Meesters, 2005). The “deep reef refugia” hypothesis, which states that mesophotic reefs may act as a refuge in the face of global reef decline (Bongaerts et al., 2010), hence might not hold for at least some Atlantic cryptochirid species.

## Supplemental Information

10.7717/peerj.1794/supp-1 Data S1 Raw data *Opecarcinus hypostegus*

10.7717/peerj.1794/supp-2 Data S2 Raw data *Troglocarcinus corallicola*

10.7717/peerj.1794/supp-3 Data S3 Raw data *Kroppcarcinus siderastreicola*

10.7717/peerj.1794/supp-4 Table S1 Locality data

## Additional Information and Declarations

### Field Study Permissions

The following information was supplied relating to field study approvals (i.e., approving body and any reference numbers): Research on Curaçao was performed under the annual research permit (48584) issued by the Curaçaoan Ministry of Health, Environment and Nature (GMN) to the CARMABI foundation.

### Data Availability

The following information was supplied regarding data availability: The raw data was supplied as Data S1–S3.
